# Enhanced X-ray diffraction of *in vivo*-grown μNS crystals by viscous jets at XFELs

**DOI:** 10.1107/S2053230X20006172

**Published:** 2020-05-29

**Authors:** Nirupa Nagaratnam, Yanyang Tang, Sabine Botha, Justin Saul, Chufeng Li, Hao Hu, Sahba Zaare, Mark Hunter, David Lowry, Uwe Weierstall, Nadia Zatsepin, John C. H. Spence, Ji Qiu, Joshua LaBaer, Petra Fromme, Jose M. Martin-Garcia

**Affiliations:** a School of Molecular Sciences, Arizona State University, Tempe, AZ 85287, USA; b Biodesign Center for Applied Structural Discovery, The Biodesign Institute, Arizona State University, Tempe, AZ 85287, USA; c Virginia G. Piper Center for Personalized Diagnostics, The Biodesign Institute, Arizona State University, Tempe, AZ 85287, USA; d Department of Physics, Arizona State University, Tempe, AZ 85287, USA; e Linac Coherent Light Source, Stanford Linear Accelerator Center (SLAC) National Accelerator Laboratory, Menlo Park, CA 94025, USA; f Eyring Materials Center, Arizona State University, Tempe, AZ 85287, USA; g ARC Centre of Excellence in Advanced Molecular Imaging, Department of Chemistry and Physics, La Trobe Institute for Molecular Science, La Trobe University, Victoria 3086, Australia

**Keywords:** μNS, avian reovirus, *in vivo* crystallization, high-viscosity jets, serial crystallography, X-ray free-electron lasers

## Abstract

μNS is a 70 kDa major nonstructural protein of avian reoviruses, which cause significant economic losses in the poultry industry. They replicate inside viral factories in host cells, and the μNS protein has been suggested to be the minimal viral factor required for factory formation. Thus, determining the structure of μNS is of great importance for understanding its role in viral infection. In the study presented here, a fragment consisting of residues 448–605 of μNS was expressed as an EGFP fusion protein in Sf9 insect cells. EGFP-μNS_(448–605)_ crystallization in Sf9 cells was monitored and verified by several imaging techniques. Cells infected with the EGFP-μNS_(448–605)_ baculovirus formed rod-shaped microcrystals (5–15 µm in length) which were reconstituted in high-viscosity media (LCP and agarose) and investigated by serial femtosecond X-ray diffraction using viscous jets at an X-ray free-electron laser (XFEL). The crystals diffracted to 4.5 Å resolution. A total of 4227 diffraction snapshots were successfully indexed into a hexagonal lattice with unit-cell parameters *a* = 109.29, *b* = 110.29, *c* = 324.97 Å. The final data set was merged and refined to 7.0 Å resolution. Preliminary electron-density maps were obtained. While more diffraction data are required to solve the structure of μNS_(448–605)_, the current experimental strategy, which couples high-viscosity crystal delivery at an XFEL with *in cellulo* crystallization, paves the way towards structure determination of the μNS protein.

## Abstract 2

The first SFX diffraction from EGFP-μNS_(448–605)_ crystals led to initial electron-density maps that allowed the clear identification of two EGFP molecules as well as of electron density for μNS_(448–605)_. Determining the crystal structure of μNS using large XFELs in combination with compact light sources will be the first step towards determining the molecular basis of the role of μNS in the early stages of virus morphogenesis.

## 1. Introduction   

Classical X-ray structure analysis requires the growth of large, well diffracting crystals, which has been a bottleneck in the process of obtaining three-dimensional structures of proteins, particularly for membrane proteins and post-translationally modified proteins. Despite the advances in ‘*in vitro*’ crystallization approaches (Gavira, 2016 ▸; McPherson & Gavira, 2014 ▸; Weselak *et al.*, 2003 ▸), namely the design of new and better sparse matrix-screening kits, the use of robotics for automated crystal formation, the development of novel crystallization methods such as counter-diffusion (Ng *et al.*, 2003 ▸) and the *in meso* crystallization method for membrane proteins (Caffrey & Cherezov, 2009 ▸), crystallization remains the major bottleneck for the structure determination of proteins.

Spontaneous protein crystallization inside living cells is a somewhat rare native process that has been known for some time (Doye & Poon, 2006 ▸). Crystallization *in cellulo* has been reported to happen in different cell organelles and is always driven by a local high protein concentration. Naturally occurring protein crystals are not accidental; *in vivo* crystal formation is associated with functions for the organism, including storage, protection, stabilization and catalysis (for a review, see Schönherr *et al.*, 2018 ▸). A recent report suggests that *in vivo* protein crystallization could be feasible for recombinant proteins (for a review, see Schönherr *et al.*, 2018 ▸). In fact, far from being the ‘holy grail’, *in vivo* crystallization offers several advantages over traditional crystallization methods such as minimizing the efforts invested in optimizing sample purification and *in vitro* crystallization, allowing the crystallization of proteins that are difficult to crystallize by conventional methods. A typical standard *in vitro* crystallization pipeline involves protein expression, purification, crystallization optimization, and crystal harvesting and cryoprotection. However, *in vivo* crystallization enables crystal growth in the cells that express the protein, bypassing the protein purification and crystallization steps (Banerjee *et al.*, 2018 ▸; Boudes *et al.*, 2016 ▸). Cells are lysed and crystals are then harvested and cryoprotected for data collection. Alternatively, the host cells are not lysed and the crystal-containing cells are delivered to the X-ray beam by standard sample-delivery methods with no need for crystal harvesting or cryoprotection (Boudes *et al.*, 2016 ▸).

Historically, the use of *in cellulo* crystals has not been deemed feasible for structural biology studies using conventional X-ray crystallography at synchrotron-radiation sources. Firstly, this is owing to the small size of crystals grown inside cells, which is frequently limited by the outer dimensions of the cell (Doye & Poon, 2006 ▸). Secondly, the crystals are of low quality and are highly sensitive to radiation damage, which is often attributable to the crowded environment in the cell that prevents the growth of sufficiently ordered crystals. However, this situation has been improved recently by the advent of the serial femtosecond crystallography (SFX) technique at X-ray free-electron lasers (XFELs) (Duszenko *et al.*, 2015 ▸; Schönherr *et al.*, 2018 ▸), along with advances in *in vivo* crystallography technology, and its adaptation as serial millisecond crystallo­graphy (SMX) on microfocus beamlines at third-generation radiation sources (Gati *et al.*, 2014 ▸). Several features, the brighter and narrower X-ray beams produced at these facilities, the advances in sample-delivery methods, much faster read-out detectors and the development of novel serial data-collection strategies, have allowed the structures of proteins to be determined from crystals in the nanometre or micrometre size range, such as those grown inside living cells. Indeed, driven by this success, the *in vivo* protein crystallization approach has been demonstrated to be a real alternative to obtaining structural information from difficult-to-crystallize proteins by applying conventional approaches. ‘*In vivo*’ crystallization was first described for structure determination by SFX at XFELs by Koopmann *et al.* (2012 ▸), with the first structure being reported by Redecke *et al.* (2013 ▸). Since then, 11 protein structures have been determined by combining these methodologies (for a review, see Schönherr *et al.*, 2018 ▸).

In the study presented here, we utilize *in cellulo* crystallization in combination with serial crystallography at XFELs to report recent advances obtained in the structural determination of the nonstructural protein μNS of the avian reovirus. Avian reoviruses (ARVs) are pathogenic viruses involved in several syndromes that are lethal to birds and cause important economic losses in the poultry industry (Jones, 2000 ▸; van der Heide, 2000 ▸). ARVs replicate in the cytoplasm of infected cells by forming so-called viral factories. These compartments, which are held together by protein–protein interactions, are thought to concentrate the required viral components to increase the overall efficiency of the replication process (Netherton *et al.*, 2007 ▸; Novoa *et al.*, 2005 ▸). The avian reovirus genome encodes 12 proteins, eight of which are structural proteins of the virion and four of which are nonstructural (NS) proteins, which are synthesized in infected cells but are not incorporated into the virus particles (Bodelón *et al.*, 2001 ▸; Tourís-Otero, Cortez-San Martín *et al.*, 2004 ▸; Varela & Benavente, 1994 ▸). Little is known about the activities and properties of most avian reovirus proteins, especially the proteins that are essential for the infection and viral replication process. Among the nonstructural proteins is μNS, a 635-residue protein of 70 kDa encoded by the M3 gene. Transfected cell studies have revealed that μNS is the minimal viral factor required for viral factory formation and that it plays an important role in the early stages of viral morphogenesis (Tourís-Otero, Cortez-San Martin *et al.*, 2004 ▸; Touris-Otero, Martínez-Costas *et al.*, 2004 ▸). The nonstructural protein μNS has also been predicted by coiled-coil predictors to have two α-helices near its C-terminus (at positions 451–472 and 540–599), which may form a coiled-coil structure (Touris-Otero, Martínez-Costas *et al.*, 2004 ▸). This structural feature has been demonstrated to be the smallest region of the protein that is necessary for globular factory formation and works by recruiting specific viral proteins to these structures (Brandariz-Nuñez *et al.*, 2010 ▸; Broering *et al.*, 2005 ▸; McCutcheon *et al.*, 1999 ▸).

To date, no structural information is available on the μNS protein; such information is crucial to better understand the mechanism by which μNS carries out its biological function. To this end, and inspired by studies performed by others (Brandariz-Nuñez *et al.*, 2010 ▸; Schönherr *et al.*, 2018 ▸), we have used a baculovirus expression-vector system to overexpress and crystallize a truncated version of the μNS protein inside Sf9 insect cells. The fragment composed of residues 448–605 was fused to an N-terminal enhanced green fluorescent protein (EGFP). Here, we describe extensive studies on the formation and biophysical characterization of the *in cellulo* crystal growth, the delivery of the crystals in viscous media to the XFEL beam and the results of the initial SFX studies at an XFEL.

## 2. Materials and methods   

### 2.1. Recombinant EGFP-μNS_(448–605)_ baculovirus generation   

To make the transfer plasmid for generation of the recombinant virus, the coding sequence of μNS (residues 448–605) was first cloned into a Gateway-compatible pIEx-nEGFP destination vector (available in the DNASU plasmid repository; https://dnasu.org/DNASU/Home.do) through one-step recombinational cloning. A transfection reaction mixture consisting of 500 ng of pEx-nEGFP-μNS_(448–605)_ transfer plasmid DNA and 100 ng of BacMagic DNA (Novagen) was incubated with 5 µl of Insect GeneJuice Transfection Reagent (Sigma–Aldrich) at room temperature for 30 min and was then added to 1 ml of Sf9 cells at a density of 1 × 10^6^ ml^−1^ maintained in suspension culture. The cell culture was incubated at 27°C and 140 rev min^−1^ for 120 h and centrifuged at 1000*g* for 5 min to obtain the supernatant containing the passage 1 (P1) recombinant EGFP-μNS_(448–605)_ viruses, followed by amplification. Briefly, 4 ml of Sf9 cells at a density of 2 × 10^6^ ml^−1^ were infected with 20 µl of P1 virus stock and incubated at 27°C and 140 rev min^−1^ for 120 h. The infected culture was centrifuged at 1000*g* for 5 min to obtain the supernatant containing the P2 virus stock.

### 2.2. Expression of EGFP-μNS_(448–605)_ in a suspension culture of baculovirus-infected Sf9 cells   

A suspension culture of Sf9 cells (Invitrogen) was maintained in Sf-900 III Serum Free Medium (Gibco) and passed to a seed density of 0.5 × 10^6^ viable cells per millilitre every other day. Cell density and viability was determined by cell staining with Trypan Blue (Invitrogen). To express EGFP-μNS_(448–605)_ fusion protein, 50 ml of Sf9 cells at a density of 1 × 10^6^ ml^−1^ were infected with 250 µl of P2 virus stock and incubated at 27°C and 140 rev min^−1^ for 72 h. A small fraction of the cell pellet was collected for protein-expression analysis by sodium dodecyl sulfate polyacrylamide gel electrophoresis (SDS–PAGE) as well as ultraviolet fluorescence (UV) microscopy.

### 2.3. Detection and verification of EGFP-μNS_(448–605)_ crystallization   

*In cellulo* crystallization of EGFP-μNS_(448–605)_ was monitored by differential interference contrast (DIC)/GFP fluorescence microscopy and SONICC (second-order nonlinear imaging of chiral crystals). At three, four and five days post-infection, a 2 µl aliquot of the suspension culture was directly sandwiched between two glass cover slides in preparation for fluorescence microscopy. The images were captured using 10×/PH2, 20×/0.50/PH2 (HCX PL Fluotar) and 40×/PH2 objectives on a Leica DM6 B motorized microscope equipped with a Leica DFC 7000T camera. Data acquisition was controlled with the *Leica Application Suite X* (*LAS X*) software. For SONICC imaging, 1 ml of the suspension culture was centrifuged at 500*g* for 5 min at 4°C. The supernatant containing the culture medium was discarded, and the insect-cell pellet was gently resuspended in 50 µl of PBS (phosphate-buffered saline; 10 m*M* Na_2_HPO_4_, 1.76 m*M* KH_2_PO_4_, 136.89 m*M* NaCl, 2.68 m*M* KCl pH 7.5) buffer. Next, 2 µl of the high-density cell suspension was loaded into a 96-well MRC 2-drop crystallization plate, sealed and immediately imaged for second-harmonic generation (SHG), which is indicative of nano/microcrystals (Wampler *et al.*, 2008 ▸), with a SONICC imager (Formulatrix; https://formulatrix.com/) using visible-light and second-harmonic generation (SHG) imaging modes. To further confirm that the crystals contained the μNS_(448–605)_ fragment, cells containing crystals were pelleted and the crystals were extracted from the cells, extensively washed and subsequently analyzed by SDS–PAGE.

### 2.4. Evaluation of the quality of EGFP-μNS_(448–605)_ crystals   

The intracellular location, size and morphology of the EGFP-μNS_(448–605)_ crystals, and crystal lattices were visualized by transmission electron microscopy (TEM). Infected insect cells were prepared following a standard TEM fixation protocol (Glauert & Lewis, 1998 ▸; Lewis *et al.*, 1977 ▸; Reid & Beesley, 1991 ▸) with modifications. For the primary fixation step, the infected insect cells were fixed using 2%(*v*/*v*) glutaraldehyde in fresh cell-growth buffer (Sf-900 III Serum Free Medium) for 15 min at room temperature followed by incubation for 2 h on ice. After washing four times (10 min each) using the cell-growth buffer and storage overnight in the same buffer, the cells were subjected to a secondary fixation step with 1%(*w*/*v*) osmium tetroxide in PBS buffer for 2 h on ice and were subsequently stained in 0.5%(*w*/*v*) aqueous uranyl acetate (UA) overnight at 4°C. Excess UA was removed by washing four times (10 min each) with deionized water (diH_2_O). Complete dehydration in acetone was followed by infiltration and embedding in Spurr’s epoxy resin. Sectioning was performed according to standard procedures. Briefly, 70 nm sections were cut using a Leica Ultracut-R microtome and collected on Formvar-coated copper slot grids. Sections were post-stained using 2% UA in 50% ethanol and Sato’s lead citrate. TEM was performed using a Philips CM 12 transmission electron microscope. Sample images were collected on a Gatan model 791 side-mount CCD camera.

### 2.5. Validation of the diffraction quality of EGFP-μNS_(448–605)_ crystals at a synchrotron source   

Three days post-infection, cells were harvested from 50 ml suspension culture by centrifugation at 500*g* for 5 min at 4°C. The cells were washed by gently resuspending them in 50 ml of PBS buffer supplemented with protease inhibitor (SigmaFAST, EDTA-Free) and centrifugation as described previously. After washing, the EGFP-μNS_(448–605)_ crystals were extracted from the Sf9 cells by gentle sonication using a Sonifier SFX550 (Branson) device operated in pulsed mode for 1 min on ice. The cycle consisted of 30 × 1 s pulses at 10% amplitude. The crystals were pelleted by centrifugation at 2000*g* for 5 min at 4°C. The crystal pellet was gently resuspended in 2 ml of PBS buffer supplemented with protease inhibitor and 30%(*v*/*v*) glycerol. Crystals were mounted on micromesh-type loops (MiTiGen) and flash-cryocooled in liquid nitrogen. X-ray data were collected on the GMCA beamline (sector 23-ID-D) at the Advanced Photon Source (APS) at Argonne National Laboratory, Chicago, USA.

### 2.6. Serial femtosecond crystallography of EGFP-μNS_(448–605)_ using viscous jets   

Upon harvesting and washing the cells as described previously, a high-density cell suspension was obtained by centrifugation at 500*g* for 5 min at 4°C and was resuspended in 2 ml of PBS buffer supplemented with protease inhibitor. SFX experiments were carried out during protein-crystal-screening (PCS) beamtime (cxip10116) in the experimental back chamber of the Coherent X-ray Imaging (CXI) instrument (Liang *et al.*, 2015 ▸) at the Linac Coherent Light Source (LCLS) at the SLAC National Accelerator Laboratory, Menlo Park, California, USA (Emma *et al.*, 2010 ▸; McNeil & Thompson, 2010 ▸). Owing to the experimental conditions required by the lead experiment in the upstream chamber, the XFEL beam was attenuated from a pulse energy of 2.5 mJ per pulse to just 40 µJ per pulse, so that the photon flux in our experiment was restricted to 2.4 × 10^10^ photons per pulse. X-ray pulses of 30 fs duration at a photon energy of 9.8 keV were focused to ∼3 µm diameter at the interaction point. The crystal number density was adjusted to approximately 2 × 10^10^ crystals per millilitre to optimize the hit rate. Two viscous media were used to deliver the crystals into the XFEL beam path: lipidic cubic phase (LCP) and agarose.

LCP-embedded crystals were prepared as described previously (Martin-Garcia *et al.*, 2017 ▸). Briefly, 5 µl of the high crystal density suspension in PBS buffer was mixed with 20 µl of molten monoolein lipid (9.9 MAG) using a dual-syringe lipid mixer (Caffrey & Cherezov, 2009 ▸; Cheng *et al.*, 1998 ▸) until a homogeneous suspension was formed. In the case of agarose, crystals were prepared as described previously (Conrad *et al.*, 2015 ▸) with some modifications. Briefly, 12%(*w*/*v*) ultralow gelling-temperature agarose (Sigma–Aldrich, catalog No. A5030) was dissolved in a solution consisting of PBS buffer and 10% PEG 400 in a 2 ml tube and heated in a thermoblock at 90°C. The agarose suspension was drawn up into a 250 µl Hamilton syringe previously warmed by drawing up and quickly ejecting boiling water a few times. The agarose suspension was then allowed to equilibrate to room temperature for approximately 20 min before 5 µl of EGFP-μNS_(448–605)_ protein microcrystals were mixed throughout the agarose using a syringe coupler (Cheng *et al.*, 1998 ▸) until the crystals were visually homogenously distributed in the agarose medium. The microcrystals embedded into the corresponding viscous medium (LCP or agarose) were loaded into 120 µl reservoirs for delivery into the XFEL beam using a high-viscosity injector (Weierstall *et al.*, 2014 ▸). The viscous medium with the crystals was extruded from the LCP injector using gas flow to form a jet of about 50 µm using a nitrogen-gas sheath. The sample-flow rate was adjusted during the experiment depending on the sample composition and the observed diffraction, with an average flow rate of 35 nl min^−1^. Single snapshots of randomly oriented crystals were recorded at a 120 Hz repetition rate using a Cornell–SLAC Pixel Array Detector (CSPAD; Hart *et al.*, 2012 ▸). The distance from the sample to the detector was set to 165 mm, corresponding to a maximum resolution of 2.5 Å at the detector edge.

### 2.7. Serial femtosecond crystallography X-ray data collection   

Peak detection and local background correction were performed using the *Cheetah* software package (Barty *et al.*, 2014 ▸). The recorded frames were then discriminated for crystal ‘hits’ based on the hit-finding parameters that define a crystal diffraction pattern (hit) by setting parameters for minimal and maximal resolution for the peak search (30 and 4 Å, respectively), minimum and maximum number of reflections (15 and 5000, respectively), minimum and maximum number of pixels per peak (1 and 20, respectively) and signal-to-noise ratio (SNR; 7). Frames that contained more than 15 detected peaks were deemed to be a hit. A total of 530 870 snapshots were recorded, 5095 of which contained single-crystal diffraction patterns (‘hits’) that were then passed to the *CrystFEL* software suite (version 0.8.0; White *et al.*, 2012 ▸, 2016 ▸) for indexing, integration and merging. Because there was no previous information about the unit cell, an initial indexing step was performed using *MOSFLM* (Powell *et al.*, 2013 ▸). This information was used in a second step to further refine the unit-cell parameters using *XGANDALF* (Gevorkov *et al.*, 2019 ▸), *MOSFLM* (Powell *et al.*, 2013 ▸), *XDS* (Kabsch, 2010 ▸), *DirAx* (Duisenberg, 1992 ▸) and *ASDF*. Crystal parameters and diffraction data statistics are summarized in Table 1 ▸.

### 2.8. Preliminary structure determination of EGFP-μNS_(448–605)_   

Merged intensities from *CrystFEL* were converted into structure-factor amplitudes using *TRUNCATE* (French & Wilson, 1978 ▸). The space group was determined by *POINTLESS *(Evans, 2006 ▸). Phase determination was carried out by molecular replacement using *Phaser* (McCoy, 2007 ▸) using the coordinates of monomer *A* of EGFP (PDB entry 3lvc; Pletneva *et al.*, 2010 ▸) as a search model. During different stages of model building and initial refinement using *Coot* (Emsley *et al.*, 2010 ▸) and *phenix.refine* (Liebschner *et al.*, 2019 ▸), respectively, the electron density was improved and two molecules of EGFP could be modeled in the asymmetric unit. The very preliminary structure of EGFP without the μNS_(448–605)_ fragment was refined at a resolution of 7 Å, with a final *R*_work_ of 34.0%. Owing to the very low resolution of our data set, refinement was carried out considering all of the reflections, so no *R*_free_ flag was applied. All illustrations were prepared using *PyMOL* version 2.3 (Schrödinger; http://www.pymol.org).

## 3. Results and discussion   

**Figure 1:**
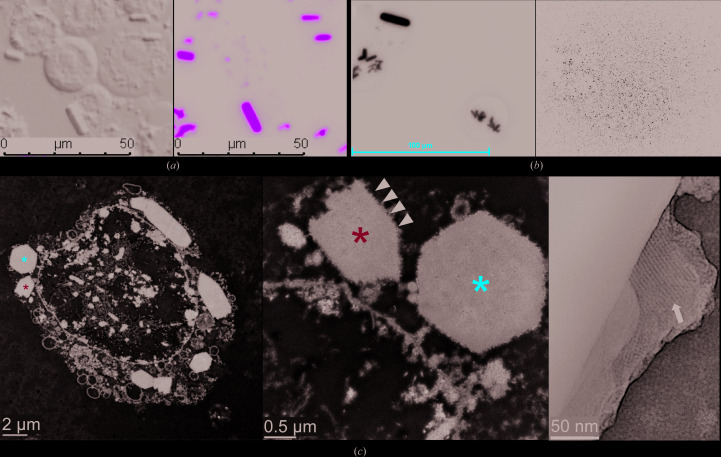
Detection and characterization of EGFP-μNS_(448–605)_ crystallization in Sf9 cells. (*a*) EGFP-μNS_(448–605)_ crystals either inside Sf9 cells or released to the medium two days post-infection observed by DIC (left) and GFP fluorescence (right) microscopy with the same field of view. (*b*) UV fluorescence image of EGFP-μNS_(448–605)_ crystals in Sf9 cells (left) and SONICC image of high-density Sf9 cells harboring EGFP-μNS_(448–605)_ crystals (right) three days post-infection. (*c*) TEM images of an Sf9 insect cell with EGFP-μNS_(448–605)_ crystals grown inside (left), EGFP-μNS_(448–605)_ crystals with a hexagonal cross section (middle) and the lattice structure of the crystal (right). Crystals denoted by asterisks in the left panel are shown in the middle panel at a higher magnification. Particles surrounding the crystals (hypothesized to be ribosomes) are indicated by black arrowheads in the middle panel. The arrow in the right panel points to the crystalline lattice.

EGFP-μNS_(448–605)_ protein was expressed and crystals were produced inside Sf9 insect cells as described by Tang (2020 ▸). Approximately 48 h after the infection of Sf9 insect cells with the recombinant baculovirus that contained the coding sequence for EGFP-μNS_(448–605)_, the formation of microstructures started to become visible. To monitor and verify EGFP-μNS_(448–605)_ crystallization in Sf9 insect cells, several imaging techniques were employed. Sf9 insect cells infected with the EGFP-μNS_(448–605)_ baculovirus displayed elongated, rod-shaped microstructures of 10–15 µm in length two days post-infection, as seen by DIC/GFP fluorescence microscopy (Fig. 1 ▸ *a*). UV fluorescence microscopy revealed that these microstructures were made up of proteins. Expression and crystallization of the whole EGFP-μNS_(448–605)_ construct was further confirmed by SDS–PAGE (Supplementary Fig. S1). Furthermore, imaging by SONICC confirmed their crystalline nature (Fig. 1 ▸ *b*). Although some crystals were observed to traverse the cell membrane (without affecting cell viability), most crystals did not exceed the normal dimensions of Sf9 cells (∼20 µm). TEM analysis further unveiled EGFP-μNS_(448–605)_ crystals that have a hexagonal cross section with sharp edges of 1–2 µm in width and consist of a well ordered crystalline lattice (Fig. 1 ▸ *c*). In addition, in the TEM micrographs particles were seen surrounding the crystals and were hypothesized to be ribosomes (Fig. 1 ▸ *c*). For a particular protein to be crystallized within the cell, a high local concentration of the protein is a prerequisite which ultimately initiates the nucleation process. In order to meet this requirement, one would expect an increasing amount of protein to be synthesized and accumulated in the cytoplasm (Duszenko *et al.*, 2015 ▸; Koopmann *et al.*, 2012 ▸). This is indeed the case during the first 48 h of expression, where the EGFP-μNS_(448–605)_ protein is present in high concentration in the cytosol, as indicated by a homogenous ‘green glow’ of the whole cytosol. However, as soon as the first crystals form the cytosol becomes ‘dark’ while the crystals still grow in size. As a result, it is possible that as soon as crystal formation occurs, protein biosynthesis of μNS by the ribosome takes place at the surface of the growing crystal. Therefore, the observed particles could be the polysome-ribosomes synthesizing μNS directly at the surface of the crystals.

During the progress of infection, the proportion of crystal-containing cells continuously increased until greater than 50% of the population contained more than one visible microcrystal per cell. However, TEM experiments showed that cells usually contain dozens of small crystals, with only a few reaching micrometre size scales (Supplementary Fig. S2). It is important to note that EGFP-μNS_(448–605)_ crystal growth occurs in the cytoplasm, as demonstrated by the intrinsic crystal fluorescence owing to the fusion protein EGFP, in agreement with previous reports (Schönherr *et al.*, 2015 ▸).

**Figure 2:**
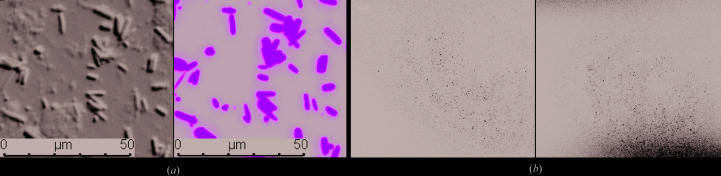
EGFP-μNS_(448–605)_ crystals resuspended in PBS buffer. (*a*) EGFP-μNS crystals extracted from Sf9 cells by gentle sonication and resuspended in PBS buffer three days post-infection observed by DIC (left) and GFP fluorescence (right) microscopy with the same field of view. (*b*) SONICC images of high-density EGFP-μNS_(448–605)_ crystal pellets extracted from Sf9 insect cells two days (left) and three days (right) post-infection.

During long-term expression of μNS_(448–605)_ over several days, an overall decrease in cell density is observed, in which cells are gradually lysed owing to the ongoing viral replication process. However, the individual EGFP-μNS_(448–605)_ crystals floating in the medium or reattached to cell remnants indicated no significant crystal damage outside the intact cell (Supplementary Fig. S3). The intrinsic stability of EGFP-μNS_(448–605)_ crystals outside the cellular environment was further evaluated after extracting the crystals from Sf9 insect cells by gentle sonication in PBS buffer pH 7.4. As confirmed by DIC/GFP fluorescence microscopy (Fig. 2 ▸ *a*) and SONICC (Fig. 2 ▸ *b*), EGFP-μNS_(448–605)_ crystals maintained their original morphology and size, with no significant degradation. The extracted crystals were incubated in PBS buffer supplemented with 30% glycerol, cryocooled, and X-ray data were collected at the APS synchrotron-radiation source. The crystals diffracted to a very low resolution of 20 Å (Supplementary Fig. S4). Despite the low resolution, sharp and well separated Bragg spots were identified, which is a significant improvement as similar crystals were previously reported to diffract to only ∼30 Å resolution (Schönherr *et al.*, 2015 ▸). The higher diffraction quality observed in our experiment compared with that of Schönherr and coworkers might be attributed to the use of cryogenic conditions, which substantially reduce radiation damage. Schönherr and coworkers collected X-ray diffraction from crystals mounted in capillaries at room temperature (Schönherr *et al.*, 2015 ▸), which very likely caused significant radiation damage to the crystals.

To increase the diffraction resolution of the EGFP-μNS_(448–605)_ crystals, we exploited highly brilliant XFEL light sources. XFELs are currently the most powerful X-ray sources, capable of producing extremely bright X-ray pulses of ultrashort duration (Emma *et al.*, 2010 ▸; Pellegrini, 2012 ▸; Pellegrini & Stöhr, 2003 ▸). Over the past ten years, SFX at XFELs has successfully been used to determine the structures of many proteins from microcrystals that only diffracted to low resolution at synchrotron-radiation sources.

Our proposal (cxip10116) for structure determination of the μNS_(448–605)_ protein was awarded protein-screening beamtime at the CXI beamline at LCLS. This PCS beamtime operates in ‘parasitic’ mode, where the lead experiment in the front chamber dictates access to the hutch as well as the beam parameters, including wavelength and photon flux. The beam passing through the hole in the detector in the front chamber is refocused before it enters the second (downstream) chamber, leading to a photon-flux reduction of approximately 50% compared with the photon flux in the upstream chamber. As described in Section [Sec sec2]2, as the experiment in the front chamber was conducted at a reduced photon flux, our experiments on the very small few micrometre-sized μNS_(448–605)_ crystals were limited to 2.4 × 10^10^ photons per pulse.

**Figure 3:**
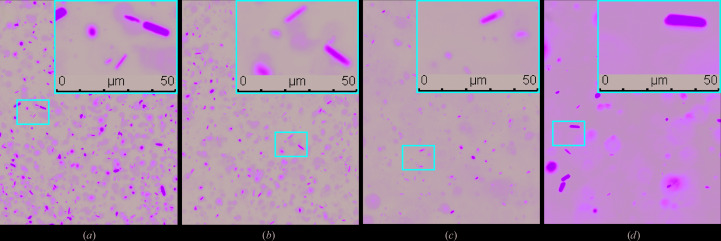
EGFP-μNS_(448–605)_ crystals embedded in LCP. GFP fluorescence microscopic images of crystals 0 h (*a*), 24 h (*b*), 72 h (*c*) and 96 h (*d*) after mixing with LCP.

In our experiment, we utilized a high-viscosity injector (Weierstall *et al.*, 2014 ▸) using two different high-viscosity sample-delivery media, LCP (Caffrey & Cherezov, 2009 ▸) and agarose (Conrad *et al.*, 2015 ▸), to deliver crystals to the ultrafast femtosecond laser pulses. Our first approach to test crystal diffraction was to embed the EGFP-μNS_(448–605)_ crystals in LCP. Before proceeding to X-ray diffraction, we evaluated the stability of the EGFP-μNS_(448–605)_ crystals upon mixing with LCP. EGFP-μNS_(448–605)_ crystals, that were released from Sf9 insect cells by gently mixing the crystal-harboring cells with LCP, exhibited significant physical stability over a time period of 0–96 h (Fig. 3 ▸) as seen by GFP fluorescence microscopy. EGFP-μNS_(448–605)_ crystals embedded and delivered in a serial fashion diffracted to 8–10 Å resolution (Supplementary Fig. S5) at LCLS. A total of 240 snapshots containing Bragg spots were recorded on the CSPAD detector, with a hit rate of 0.2%.

**Figure 4:**
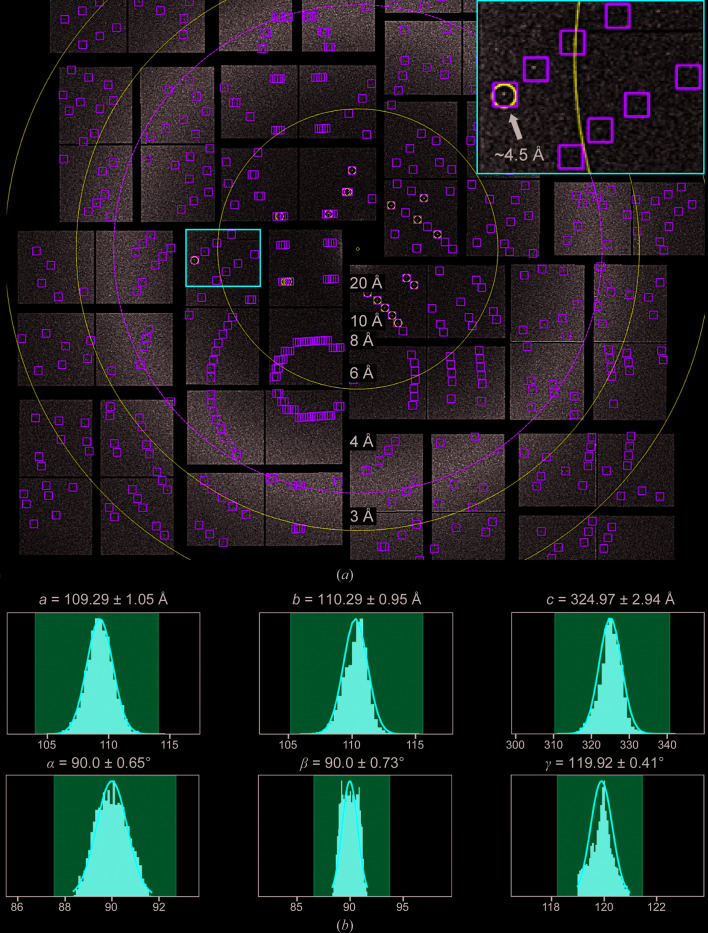
SFX of EGFP-μNS_(448–605)_ crystals delivered in agarose. (*a*) Indexed diffraction pattern of a single EGFP-μNS_(448–605)_ crystal. The black arrow in the inset shows a Bragg spot at about 4.5 Å resolution. (*b*) Unit-cell distribution of the 4227 indexed snapshots.

The second high-viscosity medium that we exploited was agarose. Agarose has recently been demonstrated to be a highly stable viscous medium and to be compatible with a wide variety of crystallization compounds, making it suitable as a crystal carrier for serial crystallography experiments (Conrad *et al.*, 2015 ▸). In our experiments, SF9 insect cells containing EGFP-μNS_(448–605)_ crystals were directly mixed with viscous medium composed of 12% agarose and 10% PEG 400 and exhibited significant physical stability as observed by polarized light microscopy (Supplementary Fig. S6). The crystals were delivered into the XFEL beam using an LCP injector. Over half a million snapshots (530 870) were recorded, with an average hit rate of 1.04%. The crystals diffracted to approximately 4.5 Å resolution (Fig. 4 ▸ *a*). Of the 5095 hits, 4227 were successfully indexed (69.8% indexing rate) in a hexagonal lattice with unit-cell parameters *a* = 109.29, *b* = 110.29, *c* = 324.97 Å (Fig. 4 ▸ *b*) and were merged in point group 6/*mmm*. The space group was determined to be *P*6_3_22 by *POINTLESS* (Evans, 2006 ▸). The diffraction images of the EGFP-μNS_(448–605)_ crystals delivered in agarose show very well defined reflections. However, the maximal resolution was potentially limited by the reduced photon flux at which the data were collected. It is conceivable that owing to the limited flux, only the larger micrometre-sized *in vivo*-grown EGFP-μNS_(448–605)_ crystals diffracted to 4.5 Å resolution, while the resolution of the smaller sub-micrometre crystals was limited to 7 Å.

Matthews analysis using the sequence of EGFP-μNS_(448–605)_ as a parameter suggested the presence of two or three molecules in the asymmetric unit, with solvent contents of 60% and 40% and Matthews coefficients (*V*_M_) of 3.07 and 2.05 Å^3^ Da^−1^, respectively (Kantardjieff & Rupp, 2003 ▸; Matthews, 1968 ▸). Phasing was carried out by molecular replacement with *Phaser* (McCoy, 2007 ▸) using the monomer from chain *A* of the structure of EGFP as a search model (PDB entry 3lvc; Pletneva *et al.*, 2010 ▸). This structure has 91% sequence identity to the EGFP sequence used in our study. Water molecules and the EGFP cofactor were removed for the analysis. The localization of the first EGFP molecule generated a single solution with a low *Z*-score and log-likelihood gain (TFZ = 5.9; LLG = 28.2). These values were significantly improved to TFZ = 15.0 and LLG = 145.6 after the localization of the second EGFP molecule. The TFZ and LLG values of 15.0 and 145.6, respectively, which are above the current minimum values aimed at by *Phaser* (TFZ = 8.0 and LLG = 120), clearly indicate that the molecular-replacement solution was found and correct. A search for a third molecule failed, which suggests that EFGP is assembled as a dimer in the asymmetric unit. Initial refinement using a resolution range between 20 and 7.0 Å with *phenix.refine* (Liebschner *et al.*, 2019 ▸) using restrained refinement with default parameters and no *R*_free_ flags applied yielded a very high* R*_work_ of 48.7%. It was necessary to include various combinations of rigid-body refinement with each of the two molecules in the asymmetric unit as one unit, simulated-annealing and *B*-factor refinement using *phenix.refine* (Liebschner *et al.*, 2019 ▸) to successfully reduce the *R*_work_ to 34.0%. It is important to note that *R*_free_ flags were not applied owing to the the extremely low number of reflections in our data set (Table 1 ▸) and the low resolution of the data set.

**Figure 5:**
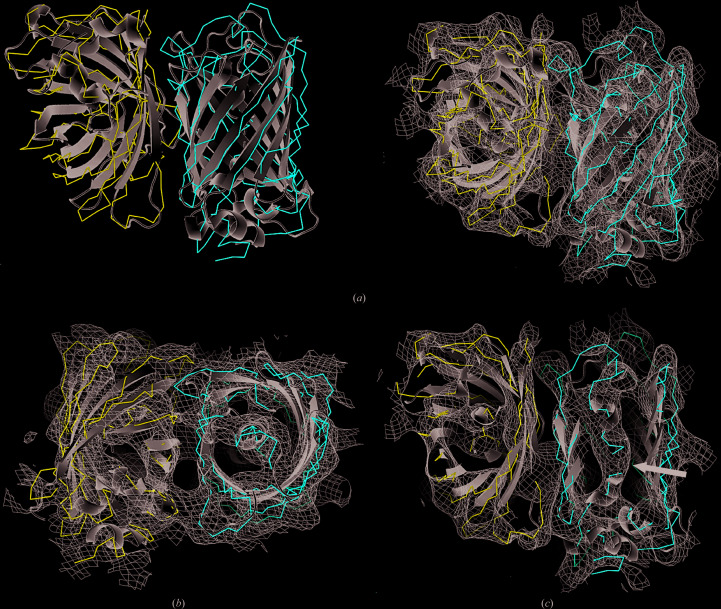
Electron-density maps (2*mF*_o_ − *DF*_c_) contoured at 1σ. (*a*) Ribbon representations of the two EGFP molecules in the asymmetric unit are shown without (left) and with (right) maps in the same orientation. (*b*) A 90° rotation of the EGFP molecules shown in (*a*). (*c*) Helical fragments running through the center of one of the EGFP molecules that coordinate the fluorescent chromophore are shown and highlighted by the black arrow. For clarity, the EGFP molecules from PDB entry 3lvc have been overlaid and are represented as a light gray cartoon in all panels shown.

**Figure 6:**
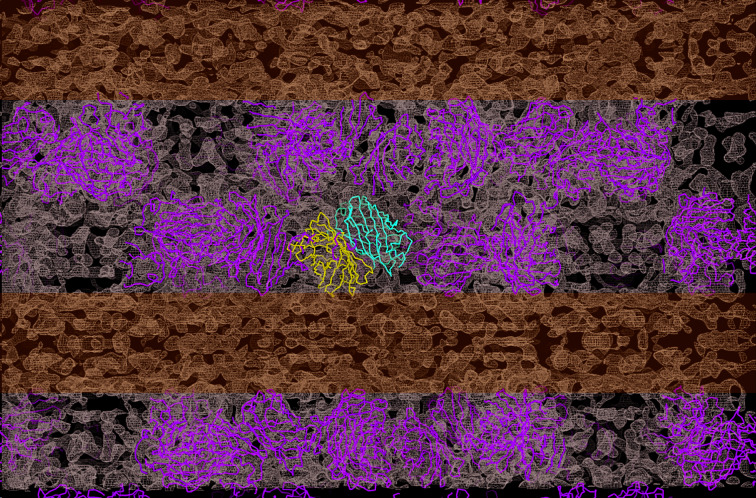
Extended electron-density maps (2*mF*_o_ − *DF*_c_). EGFP molecules in the asymmetric unit are represented as blue and red ribbons. The rest of the EGFP molecules within a 100 Å range are represented as green ribbons. The blue horizontal strips highlight the extra electron density seen between EGFP layers that is attributable to the μNS_(448–605)_ fragment.

Despite the low resolution of the data set collected, the electron density for the two molecules of EGFP can clearly be identified. The typical β-barrel topology of GFP can be identified in the electron density (Figs. 5 ▸ *a* and 5 ▸ *b*). Another detail that is also observable in our density maps is the two helix fragments running through the center of the protein to covalently coordinate the internal fluorescent chromophore (Fig. 5 ▸ *c*), which is another feature typical of all GFPs. Most importantly, we observed, for the first time, electron density that extends beyond the EGFP molecules, which belongs to the μNS_(448–605)_ fragment (Fig. 6 ▸). In an attempt to try to build a model of the μNS_(448–605)_ fragment, various tracing and model-building approaches that are currently available in both the *CCP*4 (Winn *et al.*, 2011 ▸) and *Phenix* (Liebschner *et al.*, 2019 ▸) software packages were explored unsuccessfully, which may be mainly attributed to the low resolution of the data set as well as the very weak phases for the μNS section. Also, it is important to note that no structure of μNS_(448–605)_ or a related protein exists so far (*i.e.* there is no homologous search model available), so that so the starting model for molecular replace­ment only contained the GFP section of the protein. Thus, to determine the structure of the μNS_(448–605)_ fragment from similar crystals, more SFX data need to be collected, preferably at a higher photon flux to extend the resolution of the data sets. As reported in numerous SFX publications (for a review, see Martin-Garcia *et al.*, 2016 ▸), at least 10 000 indexed diffraction patterns would be needed to accomplish this goal. As there are currently only five XFELs in operation worldwide and only one experiment can be conducted at a given time, access to XFEL beamtime is a severe bottleneck for X-ray diffraction experiments on small nanocrystals and microcrystals grown in living cells. We are therefore currently exploring whether we can further improve the *in vivo* crystal growth to obtain larger (albeit potentially fewer) crystals of μNS_(448–605)_ in the Sf9 cells, so that we can collect data on high-flux microfocus beamlines at synchrotron-radiation sources. A high-flux beamline is also under development at the ESRF in Grenoble, with a projected flux of 10^16^ photons s^−1^ and a 1 µm focus, that will be operational by the end of 2020 and might become an alternative for the collection of serial crystallography data from multiple microsized *in vivo*-grown crystals. However, as *in vivo*-grown crystals will generally always be limited in size to nanometres or up to a few micrometres, the preferred method for structure determination is SFX at XFELs.

## 4. Conclusion   

This study reports the first SFX diffraction from EGFP-μNS_(448–605)_ crystals, which led to initial electron-density maps that allowed the clear identification of two EGFP proteins as well as the identification of electron density for μNS_(448–605)_. While the interpretation of the μNS_(448–605)_ density requires more data to be collected at a higher photon flux, the results clearly indicate that SFX at XFELs using viscous jets is the method of choice to solve the structure of EGFP-μNS_(448–605)_ and likely other crystals grown *in vivo*. Our results represent an advantage in the quest to obtain higher resolution data, and it is already encouraging that the resolution reported here is the highest described to date for the μNS protein.

This method has the potential to be further extended if crystal optimization together with the use of XFEL sources can be successfully employed. XFELs have been demonstrated to be at the forefront in the structural biology field for a decade. Unfortunately, owing to their immense size (over 1 km long) and the astronomical cost of building them (over $1 billion), only five currently exist in the world. This makes beamtime applications highly competitive, and it is extremely difficult to be granted beamtime at these facilities, which hampers research in macromolecular structure solution such as, for example, obtaining the first crystal structure of μNS. Alternatively, new upcoming compact pulsed X-ray sources can also be explored. The first prototype of a compact X-ray free-electron laser is under construction at Arizona State University. This compact instrument will have a peak brilliance that is a factor of 1000 higher than that of the best conventional light sources as well as a pulse duration of 300 fs and beam sizes of a few micrometres. The performance of this unprecedented technology will significantly exceed that of current standards at the large synchrotron facilities. Thus, the use of large XFELs in combination with compact light sources to determine the crystal structure of μNS will be the first step towards determining the molecular basis of its role in the early stages of virus morphogenesis and the recruiting mechanism of specific avian reovirus proteins into viral factories through the two α-helices near the C-terminus of μNS.

## Supplementary Material

Supplementary Figures. DOI: 10.1107/S2053230X20006172/uf5001sup1.pdf

## Figures and Tables

**Table 1:** EGFP-μNS_(448–605)_ SFX data-collection statistics Values in parentheses are for the highest resolution shell.

Viscous medium	Agarose
Crystal size (µm)	5–15
Sample-to-detector distance (mm)	165
Sample flow rate (µl min^−1^)	0.035
Photon energy (keV)	9.8
Pulse duration (fs)	30
X-ray beam transmission (%)	4
Maximum resolution observed (Å)	4.5
Resolution (Å)	43.4–7.0 (7.12–7.00)
Space group	*P*6_3_22
*a*, *b*, *c* (Å)	109.29, 110.29, 324.97
α, β, γ (°)	90, 90, 120
No. of collected images	530870
No. of hits/indexed patterns/merged patterns	5095/4227/3555
Completeness (%)	100 (100)
SNR	5.1 (0.6)
CC* (%)	99.5 (70.2)
CC_1/2_ (%)	98.0 (32.7)
*R*_split_ (%)	14.8 (189.8)
Total No. of reflections	3475
No. of reflections in refinement	2023
*R*_work_ (%)	41.6
