# Supplementary material for: Enhanced X-ray diffraction of in vivo-grown μNS crystals by viscous jets at XFELs
Source: Acta Crystallogr F Struct Biol Commun. 2020 May 29;76(Pt 6):278–89. doi: 10.1107/S2053230X20006172 (PMC7278499; doi:10.1107/S2053230X20006172)
Supplement: Supplementary file 1 [file f-76-00278-sup1.pdf]

STRUCTURAL BIOLOGY COMMUNICATIONS

Volume 76 (2020)

Supporting information for article:

Enhanced X-ray diffraction of *in vivo*-grown μNS crystals by viscous jets at XFELs

Nirupa Nagaratnam, Yanyang Tang, Sabine Botha, Justin Saul, Chufeng Li, Hao Hu, Sahba Zaare, Mark Hunter, David Lowry, Uwe Weierstall, Nadia Zatsepin, John C. H. Spence, Ji Qiu, Joshua LaBaer, Petra Fromme and Jose M. Martin-Garcia

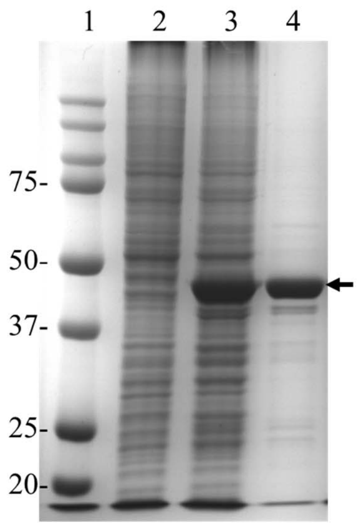


```
        10         20         30         40         50         60
MVSKGEELFT GVVPILVELD GDVNGHKFSV SGEGEGDATY GKLTLKFICT TGKLPVPWPT
        70         80         90        100        110        120
LVTTLTYGVQ CFSRYPDHMK QHDFFKSAMP EGYVQERTIF FKDDGNYKTR AEVKFEGDTL
       130        140        150        160        170        180
VNRIELKGID FKEDGNILGH KLEYNYNSHN VYIMADKQKN GIKVNFKIRH NIEDGSVQLA
       190        200        210        220        230        240
DHYQQNTPIG DGPVLLPDNH YLSTQSALSK DPNEKRDHMV LLEFVTAAGI TLGMDELYKS
       250        260        270        280        290        300
GLRSRAQASN SPAVLLSKIA DLQRANRELS LKLVDVQPAR EDHLLAYLNE HVCVNAKDHE
       310        320        330        340        350        360
KGLLARCNVS GDSISSILGQ RMKNRERFET RLRHEASAEW EPRVEALNQE LAKARVEQQD
       370        380        390        400
MMTQSLQYLN ERDELLQEVD ELKRELTTLR SANVRLNADN HRMSRATRV
```

EGFP-μNS$_{(448-605)}$	EGFP	μNS$_{(448-605)}$
409 aa	239 aa	158 aa
46.4 kDa	26.9 kDa	18.2 kDa

Figure S1 SDS-PAGE analysis of EGFP-μNS$_{(448-605)}$ crystals. 1. Molecular weight size standards (kDa). 2. Un-infected wild type Sf9 cells (negative control). 3. EGFP-μNS$_{(448-605)}$ infected Sf9 cells before crystal extraction. 4. Extracted and washed EGFP-μNS$_{(448-605)}$ crystals.

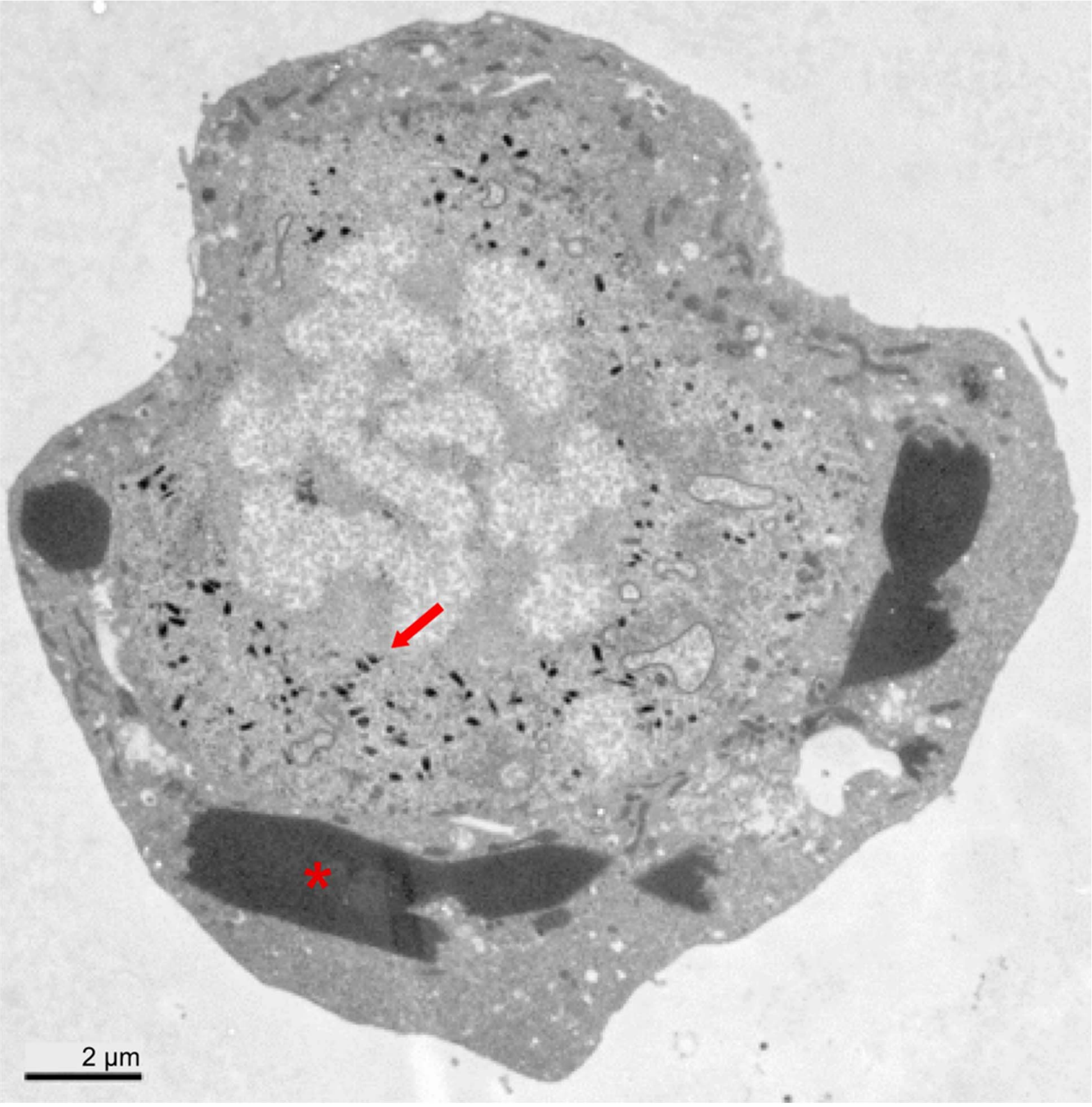

Figure S2 TEM micrograph of a single Sf9 cell harboring EGFP-µNS$_{(\Delta 1\text{-}447)}$ crystals. Among the many crystal nuclei / nano crystals formed (indicated by red arrow), only few crystals (indicated by red asterisk) reach the final dimensions of 10-15 µm in length and 1-3 µm in width.

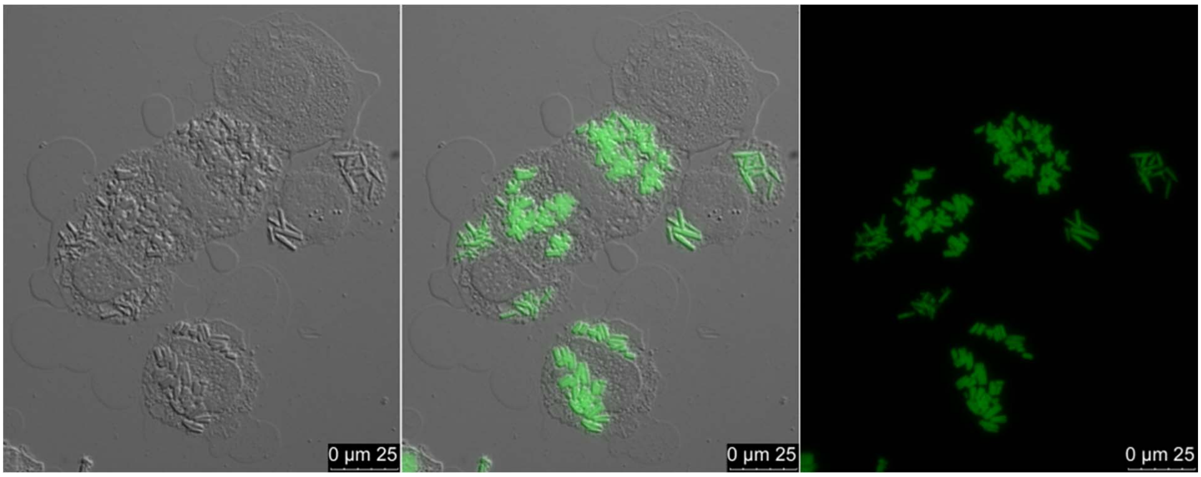

Figure S3 EGFP-µNS$_{(\Delta 1\text{-}447)}$ crystals in deteriorating Sf9 cells. Crystals are still attached to cell remnants after 4 days p.i. observed by DIC (left), DIC-GFP-fluorescence (middle) and GFP-fluorescence (right) microscopy with the same field of view.

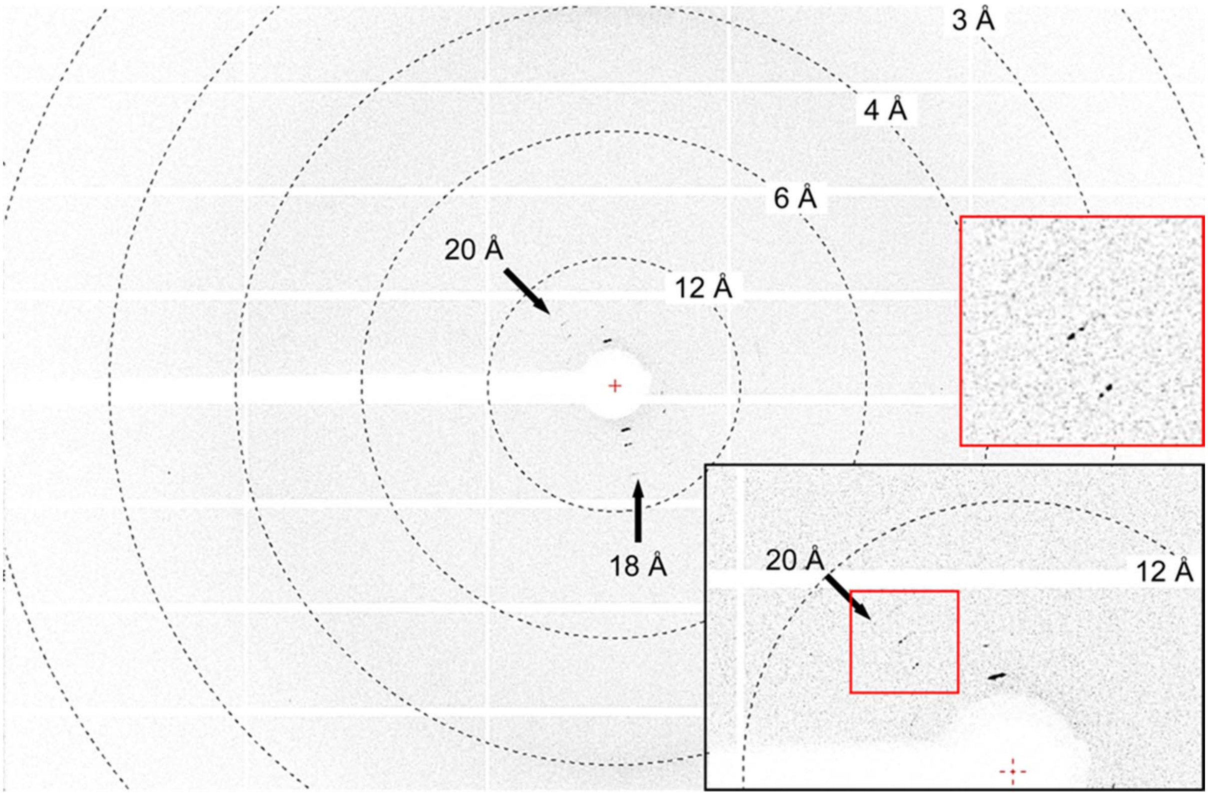

Figure S4 X-ray diffraction pattern collected from a cryo-cooled EGFP-μNS$_{(\Delta 1\text{-}447)}$ crystal. Bragg spots are seen to very low resolution as indicated by the arrows. Black boxed inset panel show a closer view of the diffracted spots. Red boxed inset panel shows the Bragg spots are well-spaced. Data were collected at GMCA 23-ID-D beamline at the Advanced Photon Source (APS).

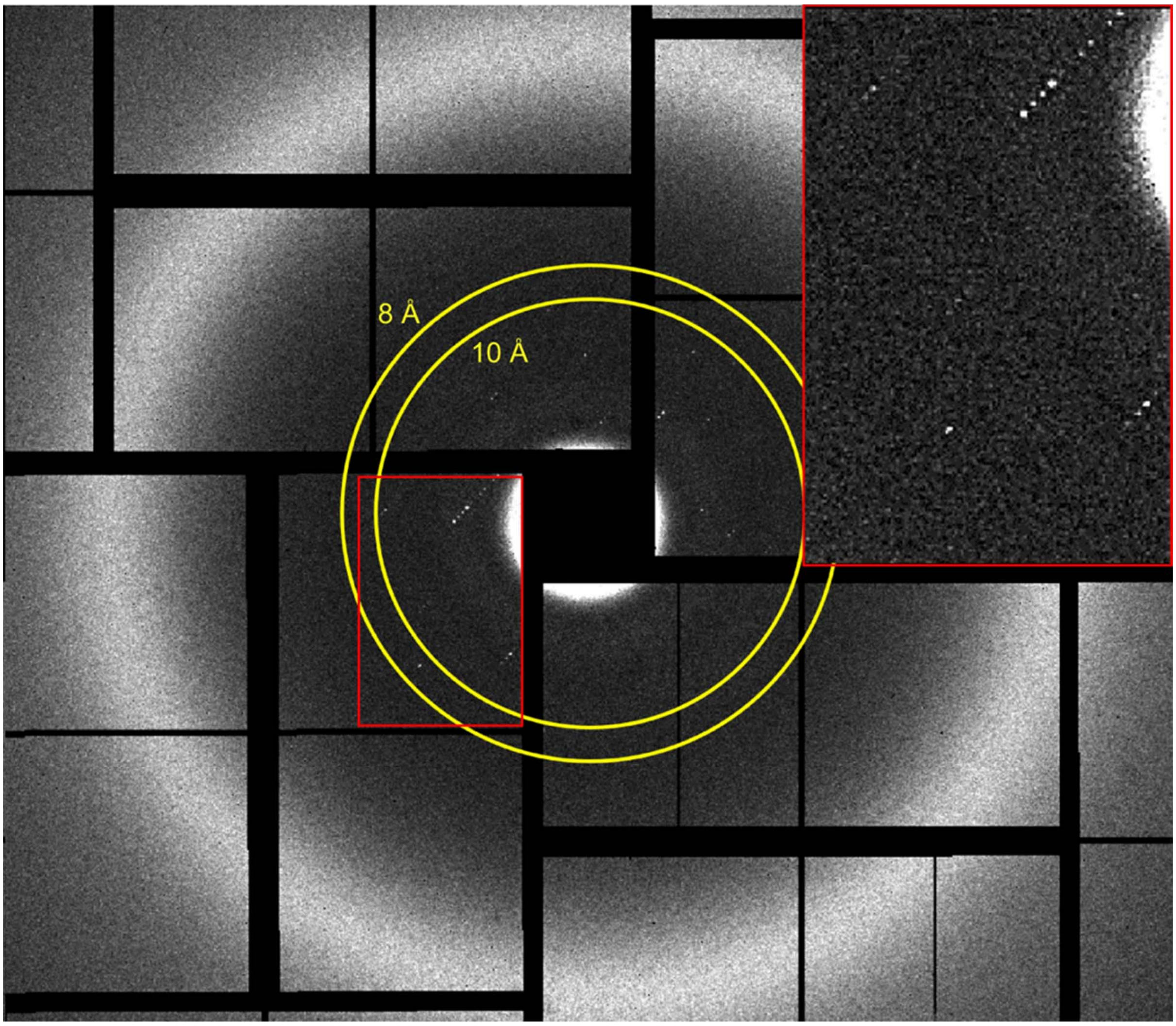

Figure S5 SFX diffraction pattern of a single EGFP-µNS$_{(\Delta 1\text{-}447)}$ crystal embedded in LCP. The inset panel shows diffraction to ~10Å. Small separation between spots suggests a big unit cell. The typical LCP ring appears as a white aureole to about 4.5Å.

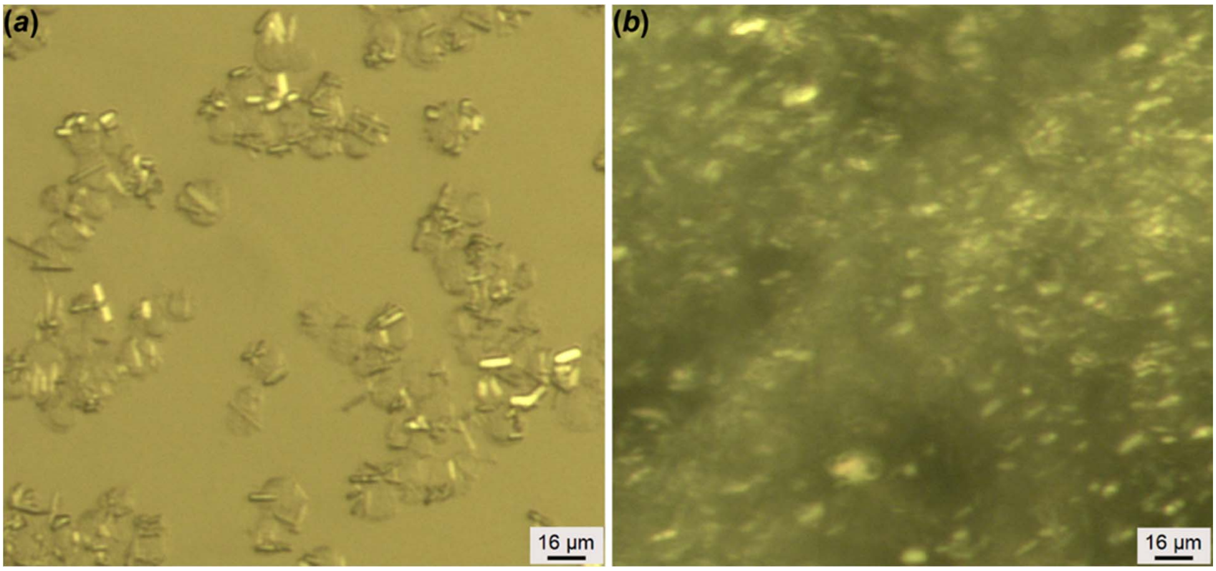

Figure S6 EGFP-µNS$_{(\Delta 1\text{-}447)}$ crystals embedded in agarose. Sf9 insect cells harboring EGFP-µNS$_{(\Delta 1\text{-}447)}$ crystals were harvested and stored in PBS for 5 - 7 days at 4°C until X-ray diffraction. Cross-polarized light images before *(a)* and after *(b)* mixing with agarose.
